# Ectoin attenuates cortisone‐induced skin issues by suppression GR signaling and the UVB‐induced overexpression of 11β‐HSD1

**DOI:** 10.1111/jocd.16516

**Published:** 2024-09-02

**Authors:** Dailin Xu, Yue Wu

**Affiliations:** ^1^ In Vitro Research Department Bloomage Biotechnology Corporation Limited Shanghai China

**Keywords:** cortisol, cortisone, Ectoin, skin issues, stress

## Abstract

**Background:**

Accelerated pace of modern work and lifestyles subject individuals to various external and psychological stressors, which, in turn, can trigger additional stress through visible signs of fatigue, hair loss, and obesity. As the primary stress hormone affecting skin health, cortisol connects to the glucocorticoid receptor (GR) to aggravate skin issues induced by stress. This activation depends on the expression of 11β‐hydroxysteroid dehydrogenase 1 (11β‐HSD1) in skin cells, which locally converts cortisone—produced by the central and peripheral hypothalamic‐pituitary‐adrenal axis—into its active form.

**Methods:**

Our study delves deeper into stress's adverse effects on the skin, including the disruption of keratinocyte structural proteins, the loss of basement membrane proteins, and the degradation of collagen.

**Results:**

Remarkably, we discovered that Ectoin, an amino acid derivative obtained from halophilic bacteria, is capable of mitigating the inhibitory impacts of cortisone on the expression of cutaneous functional proteins, including involucrin, loricrin, laminin‐5, and claudin‐1. Moreover, Ectoin reduces the suppressive effect of stress on collagen and hyaluronic acid synthesis by impeding GR signal transduction. Additionally, Ectoin counterbalances the UVB‐induced overexpression of 11β‐HSD1, thereby diminishing the concentration of endogenous glucocorticoids.

**Conclusion:**

Our findings illuminate the significant potential of Ectoin as a preventative agent against stress‐induced skin maladies.

## 1 INTRODUCTION

The human skin is the largest organ in the human body, also served as the “boundary” between the body and the environment. Unlike other tissues and organs, the skin is endowed with the innate ability to “sense,” “process,” and “react” when constantly exposed to a variety of stressors.^1^ Stress factors, including psychological aspects^2^, ^3^ (e.g., pain, anxiety, depression), environmental influences^4^, ^5^ (e.g., pollution, UV and blue light exposure, extreme weather conditions), and social pressures^6^, ^7^, ^8^ (e.g., examinations, lack of sleep, nocturnal habits, high‐pressure work), all of which can activate the hypothalamic‐pituitary‐adrenal (HPA) axis and then trigger a systemic stress reaction in the body.

Stress, stemming from fluctuations in internal and external environments, necessitates a rapid response from our bodies. However, prolonged exposure to stress can upset this delicate equilibrium, rendering the body more vulnerable to stress‐related illnesses.^9^, ^10^, ^11^ The effects of stress vary across different age groups and social levels, and even within the same demographic, stressors are distinctively shaped by individual personality traits. Stress is a recognized trigger for the initiation, aggravation, and recurrence of numerous skin conditions,^6^ including itchiness, nodular prurigo, atopic dermatitis, psoriasis, urticaria, alopecia areata, and telogen effluvium.^2^, ^12^, ^13^ Glucocorticoids (GCs), produced in response to stress, can alter the activity of various skin cells, leading to negative consequences such as alterations in skin texture and a damaged skin barrier. Manifestations of such outcomes including broken epidermal layer integrity, weakened dermal‐epidermal junction (DEJ), anti‐anabolic metabolism in dermal fibers, and diminished immune function.^14^, ^15^, ^16^, ^17^ Crucially, GCs exposure and glucocorticoid receptor (GR) activation can cause long‐lasting epigenetic modifications, most notably DNA methylation, in addition to acute alterations in gene expression.^18^

Cortisol, is commonly regarded as one of the primary GC hormone secreted in response to HPA axis. It is necessary for the metabolism of proteins, fats, and carbohydrate as well as modulating the immune response.^19^ In addition, cortisol affects mood, behavior, neuroendocrine function, temperature regulation, and perception of pain. Under normal physiological conditions, approximately 90% of cortisol in the bloodstream binds to corticosteroid‐binding globulin (CBG). On the other side, circulating cortisone—the inactive state of cortisol—has a lower affinity for CBG,^5^ thus converted into active cortisol by 11β‐hydroxysteroid dehydrogenase type 1 (11β‐HSD1) located in the tissues. In times of stress, due to blood's limited binding capacity, a greater proportion of cortisol circulates in its unbound, inactive form, elevating local tissue GC levels. Moreover, the skin itself harbors a peripheral HPA axis, capable of secreting numerous neuropeptides such as CRH, POMC, oxytocin, and cortisone.^12^ The 11β‐HSD1 locally modulates the availability of active GCs to GRs.^20^ Its expression is detected in various cell types including those across all epidermal layers (except stratum corneum), dermal fibroblasts, and sebaceous and sweat gland cells.^21^, ^22^, ^23^ An upregulation of 11β‐HSD1 has been correlated with the impairment of oral mucosal barrier function and the delayed healing of skin wounds.^6^, ^14^ This suggests that the expression levels of 11β‐HSD1 at different sites may directly influence the intensity of active cortisol's effects.

Previous research has demonstrated the stress‐induced harmful impacts on the skin, herein, we attempt to elucidate alterations in functional molecules within a cortisone‐induced skin stress cellular model. Our investigations led us to identify Ectoin, an amino acid derivative isolated from the halophilic bacterium Halomonas elongata, within our library of bioactive compounds. Ectoin regulates cellular osmotic pressure and serves as a natural “protective shield,” enabling halophilic bacteria to thrive in harsh environments characterized by extreme heat, salinity, and intense ultraviolet (UV) radiation. Past research has highlighted Ectoin's remarkable abilities in water retention and protective capabilities for skin care and cell structures.^24^, ^25^, ^26^, ^27^ Within this paper, we attempt to explore the potential ability of Ectoin to mitigate cortisone‐induced skin issues within an artificial stress environment and to detail the underlying mechanisms at play.

## 2 MATERIALS AND METHODS

### 2.1 Cell culture and reagent

Normal human dermal fibroblasts (NHDF) were obtained from the American Typical Culture Collection Center and cultivated in 10% fetal bovine serum (FBS; Gibco) and 1% streptomycin–penicillin solution (Gibco), supplemented with high‐glucose Dulbecco's Modified Eagle's Medium (Gibco), at 37°C, and 5% CO_2_. Immortalized human epidermal (HaCaT) cells were obtained from QingQi (Shanghai) Biotechnology Company and cultivated in 10% FBS (Gibco) and 1% streptomycin–penicillin solution (Gibco), supplemented with high‐glucose Dulbecco's Modified Eagle's Medium (Gibco), at 37°C, and 5% CO_2_. Subcultured cells were utilized for additional tests after they reached 80% confluency.

All reagents without specifically stated were from sigma‐aladin (St Louis, MI, USA).

### 2.2 Cell viability assay

MTT (3‐[4,5‐dimethylthiazol‐2‐yl]‐2,5‐diphenyltetrazolium bromide), acquired from VWR Corp., Ohio, USA, was used to assess the vitality of the cells. After plating the NHDF and HaCaT cells in 96‐well plates, they were incubated for the whole night. The cells were then subjected to various dosages Ectoin of 0, 0.0625, 0.125, 0.25, 0.5, and 1 mg/mL diluted with 2% FBS DMEM treatment at 37°C, 5% CO_2_ in a cell culture incubator. Subsequently, each well was filled with 0.5 mg/mL of MTT reagent, and the cells were incubated for 3 h. After that, the media was thrown away and DMSO was used to solubilize the cells. The absorbance was determined with a spectrophotometer set at 550 nm in wavelength.

### 2.3 Immunocytochemistry

Loricrin (LOR), Involucrin (INV), Hyaluronan synthase 2 (HAS2), Claudin‐1 (CLDN1), Laminin‐5 (LN5), Collagen I (COLI), were determined by immunocytochemistry.

Typically, 1.2 × 10^4^ NHDF cells or 3 × 10^4^ HaCaT cells were plated in 24‐well plates, cultured for 24 h, and then treated with cortisone (100 μM) for 24 h with or without Ectoin (0.5 mg/mL). The cells were permeabilized with 0.1% triton X‐100, fixed in 4% formaldehyde, and then treated with primary antibody for the whole night at 4°C. The cells were washed before being treated for 1.5 h with secondary antibodies matching the species. Finally, the Leica DM IL LED inverted microscope was used to study the cells.

### 2.4 Quantitative PCR

Plated in 6‐well plates, NHDF cells (6 × 10^5^/well) were cultivated for a duration of 24 h. Following a PBS rinse, the cells were incubated for 12 h in a serum‐free medium containing 100 μM cortisone and either vehicle or 0.5 mg/mL Ectoin. Following this therapy, TRIzol reagent (Invitrogen, Carlsbad, CA, USA) was used to extract total RNA samples from the cells, and a Micro spectrophotometer (SMA4000; Merinton Instrument, Inc.) was used to quantify the samples. Next, in accordance with the manufacturer's recommendations, an equivalent volume of each RNA sample was used for cDNA synthesis using Maxima Reverse Transcriptase (Thermo Scientific). Using a CFX Real‐Time System (Bio‐Rad), quantitative PCR was performed in a final volume of 20 μL, which included 10 μL of the Universal SYBR Green Supermix (Bio‐Rad), 0.6 μM of each primer, and 50 ng of template cDNA. To normalize gene expression, GAPDH was used as a reference gene. Each gene's primer sequences are reported in Table S1.

### 2.5 Western blotting

In T25 cell culture flasks, NHDF cells (1 × 10^6^/well) were seeded and cultured for a duration of 24 h. Every media containing serum was taken out and replaced with medium devoid of serum. The cells were then cultivated for 30 min with either a vehicle or 0.5 mg/mL of Ectoin in the presence of 100 μM cortisone. Subsequently, lysing the cells with a protein extraction solution (PIPA, P0013B) was done. Proteins in the lysates were separated by sodium dodecyl sulfate polyacrylamide gel electrophoresis and transferred to a nitrocellulose membrane (Whatman, Dassel, Germany) following centrifugation at 13000× g for 20 min at 4°C. After 2 h of incubation in tris‐buffered saline containing 0.05% Tween 20 and 5% BSA, the membranes were left to overnight at 4°C to be treated with primary antibodies. Abcam (Cambridge, UK) supplied the primary antibodies against the following proteins: GR (ab183127), p‐GR (p‐GR; ab55189), and the primary antibody against GAPDH (ab8245). After that, the membranes were probed for 1 h using a secondary antibody that was coupled to the IgG of either mice or rabbits (Abcam, Cambridge, UK). Reaction product visualization was accomplished using the Tanon 6600 luminescence imaging workstation. The Imagen Pro Plus 6.0 was utilized for densitometry in order to quantify the relative band densities. The loading control was GAPDH.

### 2.6 Statistical analysis

The findings are presented as mean ± SD, with statistical significance being determined by *p*‐values less than 0.05. With the use of Dunnett's posttest and one‐way analysis of variance, GraphPad 9.5.0 (GraphPad Software, San Diego, CA, USA) was used.

## 3 RESULTS

### 3.1 Ectoin has no effect on the viability of human dermal fibroblasts and human immortalized epidermal cells

**FIGURE 1:**
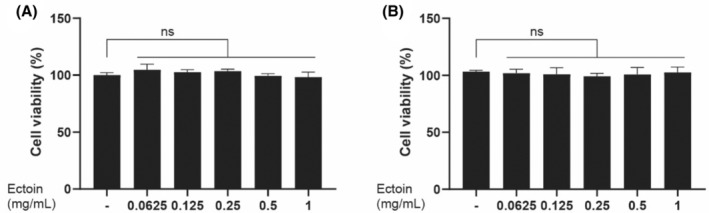
Effect of Ectoin on the viability of human dermal fibroblasts (A) and human immortalized epidermal (HaCaT) cells. (B) The cells were exposed to various dosages of Ectoin (0.0625, 0.125, 0.25, 0.5, and 1 mg/mL) or the vehicle (ultrapure water; indicated by the symbol “‐”) for a duration of 72 h. The MTT test was used to measure cell viability. The following expressions are used to indicate statistical significance: Ns *p* > 0.05.

To evaluate the potential effect of Ectoin on cell viabilities, we performed an MTT assay on NHDF and HaCaT cells. Our findings revealed that Ectoin, at concentrations as high as 1 mg/mL, had no effect on the survival of either cell type (Figure 1A,B).

### 3.2 Ectoin attenuates cortisone‐induced suppression of skin barrier functions both on epidermal and dermal skin cells

The skin serves as the body's primary protective shield, safeguarding against external chemical and physical threats, engaging in metabolic processes, and sustaining homeostasis. In our study, we aimed to determine whether Ectoin can alleviate skin barrier impairment under stressful conditions by initially assessing the barrier function in epidermal skin cells.

**FIGURE 2:**
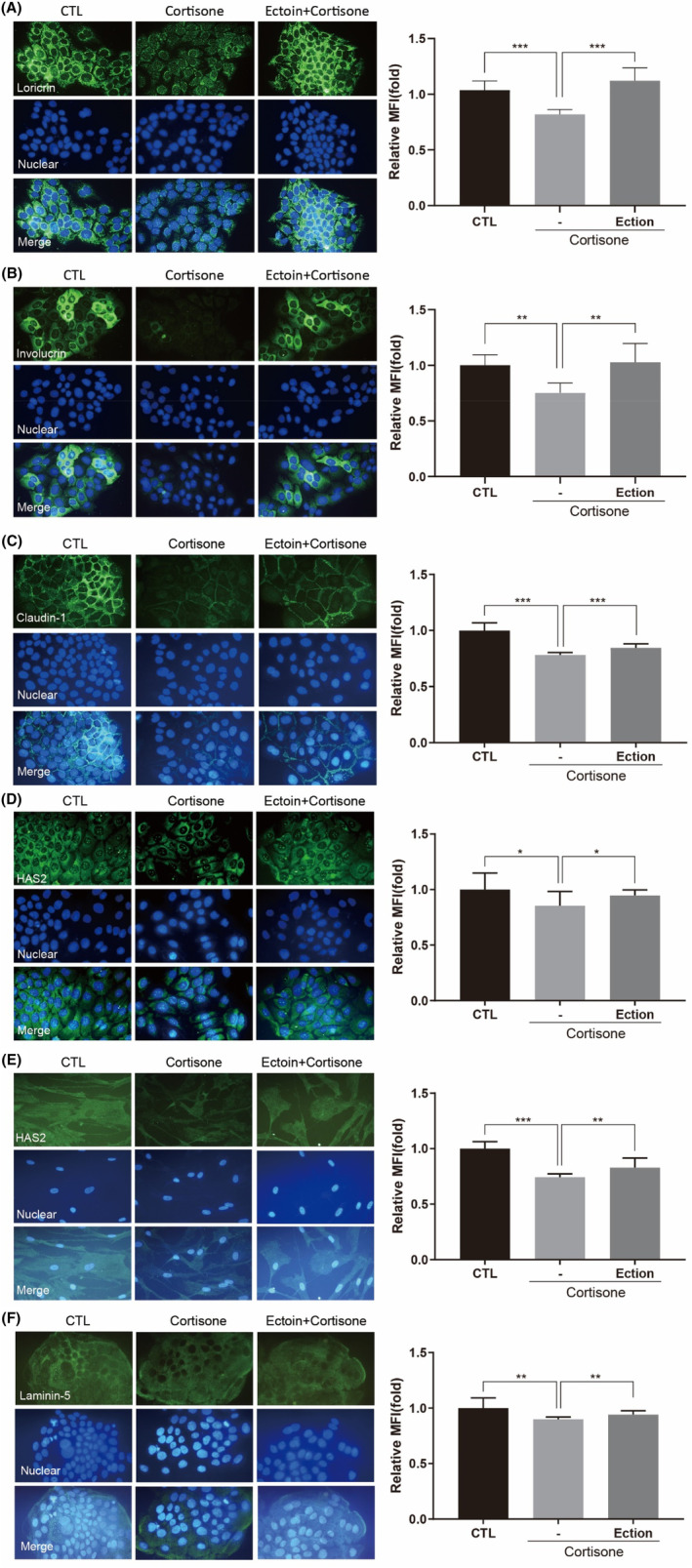
Effect of Ectoin on skin barrier functions associated biomarkers expression under cortisone treatment in HaCaT and NHDF cells. Treatment of the cells were either vehicle (labeled “CTL”) or cortisone (100 μM) with or without Ectoin (0.5 mg/mL) for 72 h. (A) Loricrin (LOR), (B) Involucrin (INV), HAS2 in HaCaT cells, (C) and Normal human dermal fibroblasts (NHDF) (D, E) Claudin‐1 and (F) Laminin‐5 expression were measured by immunofluorescence staining. Data are reported as the mean ± SEM across the three experiments. The following is how statistical significance is expressed: *p < 0.05, **0.01, and ***0.001.

Treatment with cortisone markedly suppressed the formation of cornified envelope, a structure predominantly composed of LOR and INV. However, Ectoin was found to enhance the expression of LOR and INV in HaCaT cells, under the treatment of cortisone (Figure 2A,B). Moreover, the integrity of tight junction (TJ)^28^, ^29^ on epidermal is essential for maintaining the epidermal selective permeable barrier functions. Ectoin attenuates cortisone‐induced suppressed of CLDN1, a TJ structural protein (Figure 2C). Consequently, these findings suggest that Ectoin may help preserve the functional integrity of the keratinocyte barrier, potentially protecting the skin from stress‐induced impairment.

HAS2 catalyzed the high molecular weight hyaluronic acid (HA) synthesis in skin, which playing a greatly role in maintaining skin physiological structure and barrier homeostasis. Next, we measured HAS2 production in cortisone treated human HaCaT and NHDF cells by immunofluorescent staining. Our findings indicate that cortisone significantly reduced HAS2 expression in both HaCaT (Figure 2D) and NHDF (Figure 2E) cells. Importantly, the decrease in HAS2 expression caused by cortisone was substantially reversed by the presence of Ectoin, suggesting its potential in restoring skin's barrier function.

LN5,^30^, ^31^, ^32^ critical protein of the DEJ, is crucial for tissue repair, cell adhesion, migration, and skin integrity. Finally, we measured the expression of LN5 in cortisone‐treated HaCaT cells. Cortisone substantially decreased LN5 production in comparison with control groups. In contrast, Ectoin treatment was found to increase the production of LN5 (Figure 2F), suggesting a supportive role for Ectoin in maintaining DEJ protein levels.

In summary, Ectoin has been demonstrated to protect the skin barrier from stress by preserving the normal functionality of cells across different skin layers to a significant degree.

### 3.3 Ectoin attenuates cortisone‐induced skin aging on dermal skin cells

Skin texture alterations are, to some extent, linked to the natural aging process, a phenomenon that stress is known to expedite.^33^ This phenomenon is exemplified by the irreversible skin atrophy observed in patients undergoing long‐term GC therapy.^34^, ^35^ Increased excessive matrix metalloproteinase‐1 (MMP‐1) secretion and reduced type I expression are the main characteristics of this illness.

**FIGURE 3:**
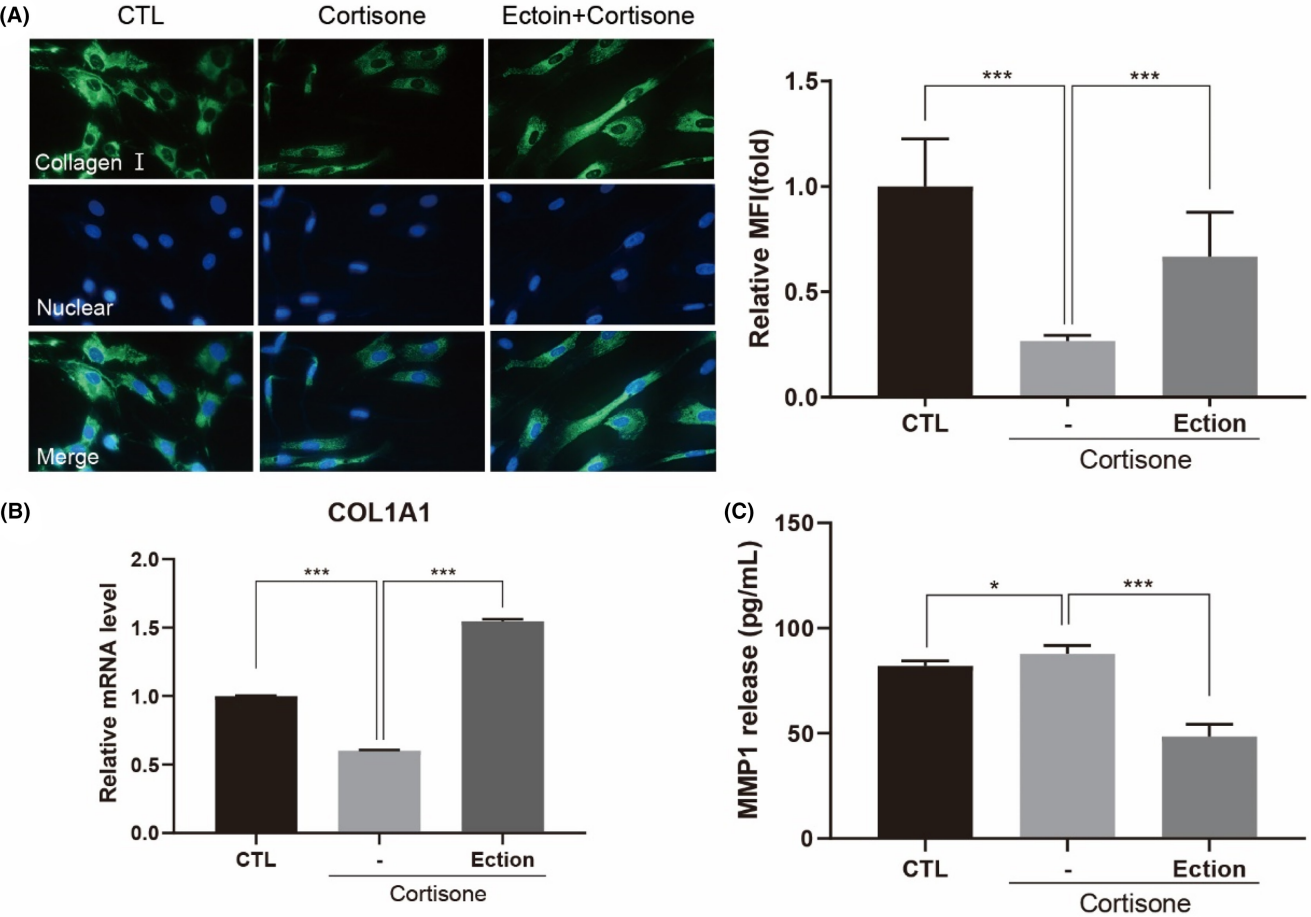
Impact of Ectoin on collagen synthesis and MMP‐1 content in NHDF. Treatment of the cells were either vehicle (shown as “CTL”) or cortisone (100 μM) with or without Ectoin (0.5 mg/mL) and for 72 h. (A) Collagen I expression were measured by immunofluorescence staining in human dermal fibroblasts, and (B) MMP‐1 levels was quantitated by MMP‐1 Elisa kit. Data are reported as the mean ± SEM across the three experiments. The following is how statistical significance is expressed: *p < 0.05 and ***0.001.

To assess whether Ectoin enhances COLI synthesized in cortisone‐treated fibroblasts, we measured COLI expression both on the levels of gene (collagen type I alpha 1, COL1A1) and protein. COLI production was significantly reduced after cortisone treatment, but notably, Ectoin counteracted this cortisone‐induced decrease in COLI (Figure 3A). Consistent with this result, Ectoin also markedly upregulated the gene expression levels of COL1A1 (Figure 3B). Moreover, Ectoin mitigated the oversecretion of MMP‐1 induced by cortisone (Figure 3C), which otherwise protected collagens from GCs induced anti‐anabolic effect.

### 3.4 Ectoin inhibits UVB‐induced 11β‐HSD1 overexpression

Activation of cortisol within the skin is linked to 11β‐HSD1. Research on human skin tissue explants has shown that exposure to light increase the expression of 11β‐HSD1.^36^ Psychological stress in deteriorative state has been linked to increased cortisol levels in the epidermal layer and impaired barrier function, which are directly correlated with elevated 11β‐HSD1 activity.^37^, ^38^ Similarly, aging skin shows an impaired epidermal permeability barrier, which is also tied to increased amounts of 11β‐HSD1.^5^ In Section 3.2, it was shown that Ectoin could mitigate the impact of cortisone on the structural integrity of cornified envelope and the basement membrane, both integral to skin barrier conditions. Building on this finding, we delved deeper into the role of Ectoin as a potential inhibitor of 11β‐HSD1 expression.

**FIGURE 4:**
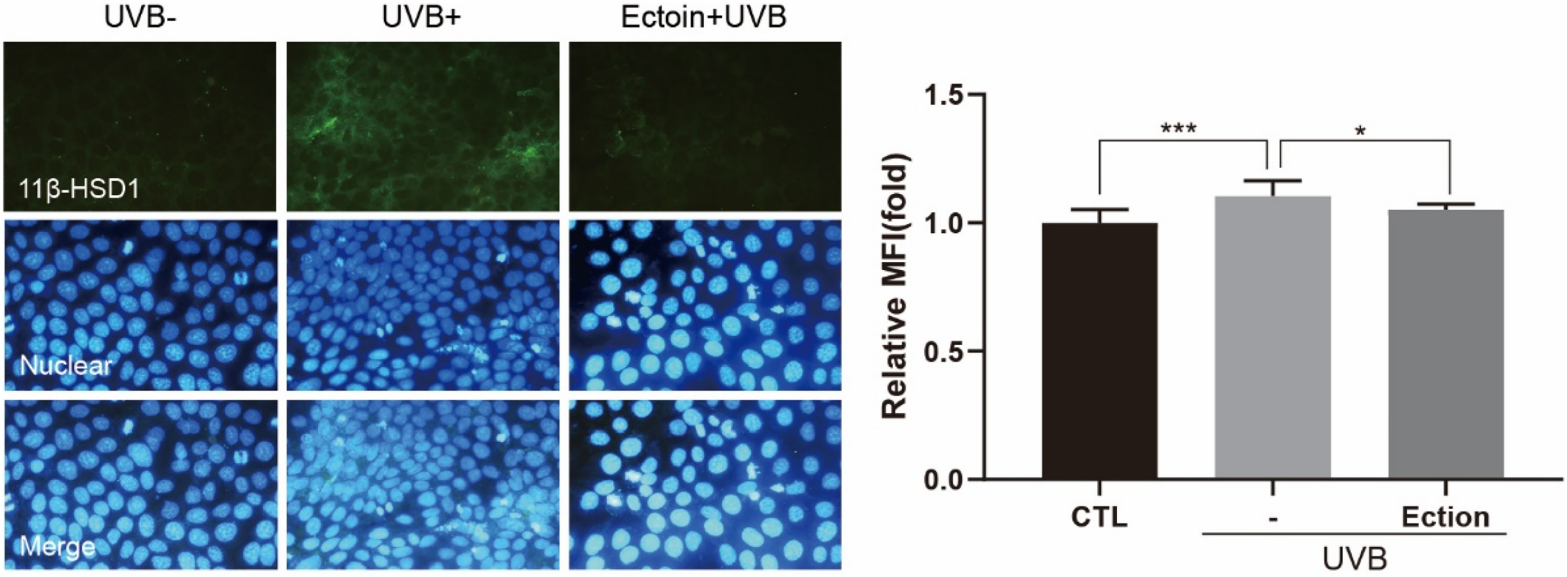
Ectoin inhibited the increase of 11β‐HSD1 in UVB‐exposed HaCaT cells. (A) 11β‐HSD1 in protein level were measured by Immunofluorescence staining. Treatment of HaCaT cells were 0.5 mg/mL of Ectoin for 24 h, and cells were exposed to UVB (5 mJ/cm^2^) before the treatment. The following is how statistical significance is expressed: *p < 0.05 and ***0.001.

Our preliminary investigation showed that UVB radiation led to the overexpression of 11β‐HSD1 in HaCaT cells (Figure S1). We selected a dose of 5 mJ/cm^2^ for irradiation, under which conditions no cytotoxicity was observed (Figure S2). After UVB irradiation, HaCaT cells showed a heightened 11β‐HSD1 protein level compared to the control group. However, treatment with Ectoin succeeded in reducing 11β‐HSD1 expression (Figure 4). Evidence suggests that Ectoin possesses a potent protective effect against the overexpression of 11β‐HSD1 induced by UVB radiation. This finding provides a defensive measure to mitigate the negative effects of stress on skin exposed to light.

### 3.5 Mechanism exploration on Ectoin inhibits cortisone‐induced skin issues

Cortisone, a small steroid hormone, is capable of readily diffusing from dermal capillaries into the skin. Once inside the skin, cortisone is converted to its active form, which is cortisol, via 11β‐HSD1, which is located on the endoplasmic reticulum. Active cortisol subsequently binds to the GRs within the cytoplasm. The ligand‐bound GR complex subsequently translocate to the nucleus, where it modulates the expression of target genes through a variety of mechanisms, including transactivation, transrepression, and non‐genomic signaling pathways.^39^

**FIGURE 5:**
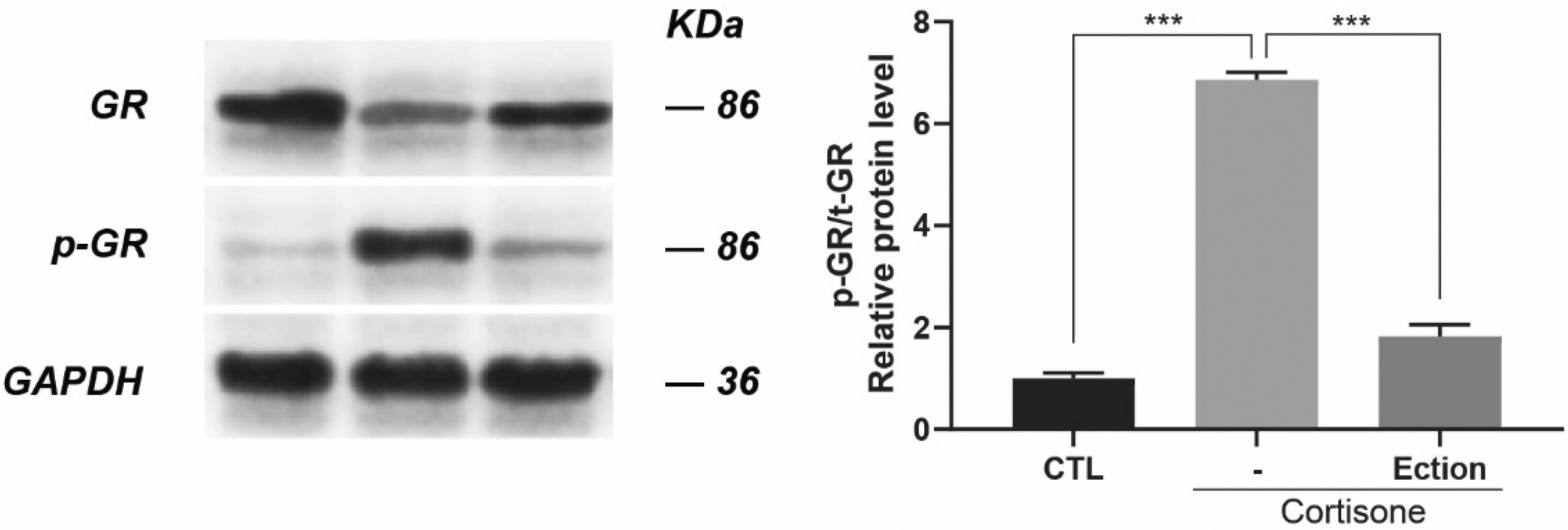
Effect of Ectoin on GR signaling. Treatment of the cells were either vehicle (labeled as “CTL”) or cortisone (100 μM) with or without Ectoin (0.5 mg/mL) for 30 min. Western blotting was used to create and examine whole‐cell protein extracts. After normalizing both the total and phosphorylated GR levels to the GAPDH level, the phosphorylated/total GR ratio was computed. The ratio of phosphorylated GR to total GR is presented (p‐GR/t‐GR). The following is how statistical significance is stated: ***0.001.

To determine whether Ectoin interrupts cortisone‐induced GR signaling pathway, thereby alleviating cortisone‐induced skin issues, we measured GR phosphorylation. We analyzed the ratio of phosphorylated to total GR following normalization to GAPDH through densitometry of protein bands. Our results showed cortisone‐induced GR phosphorylation in NHDF, while conversely, Ectoin notably inhibited cortisone‐induced GR phosphorylation in NHDF cells (Figure 5).

**FIGURE 6:**
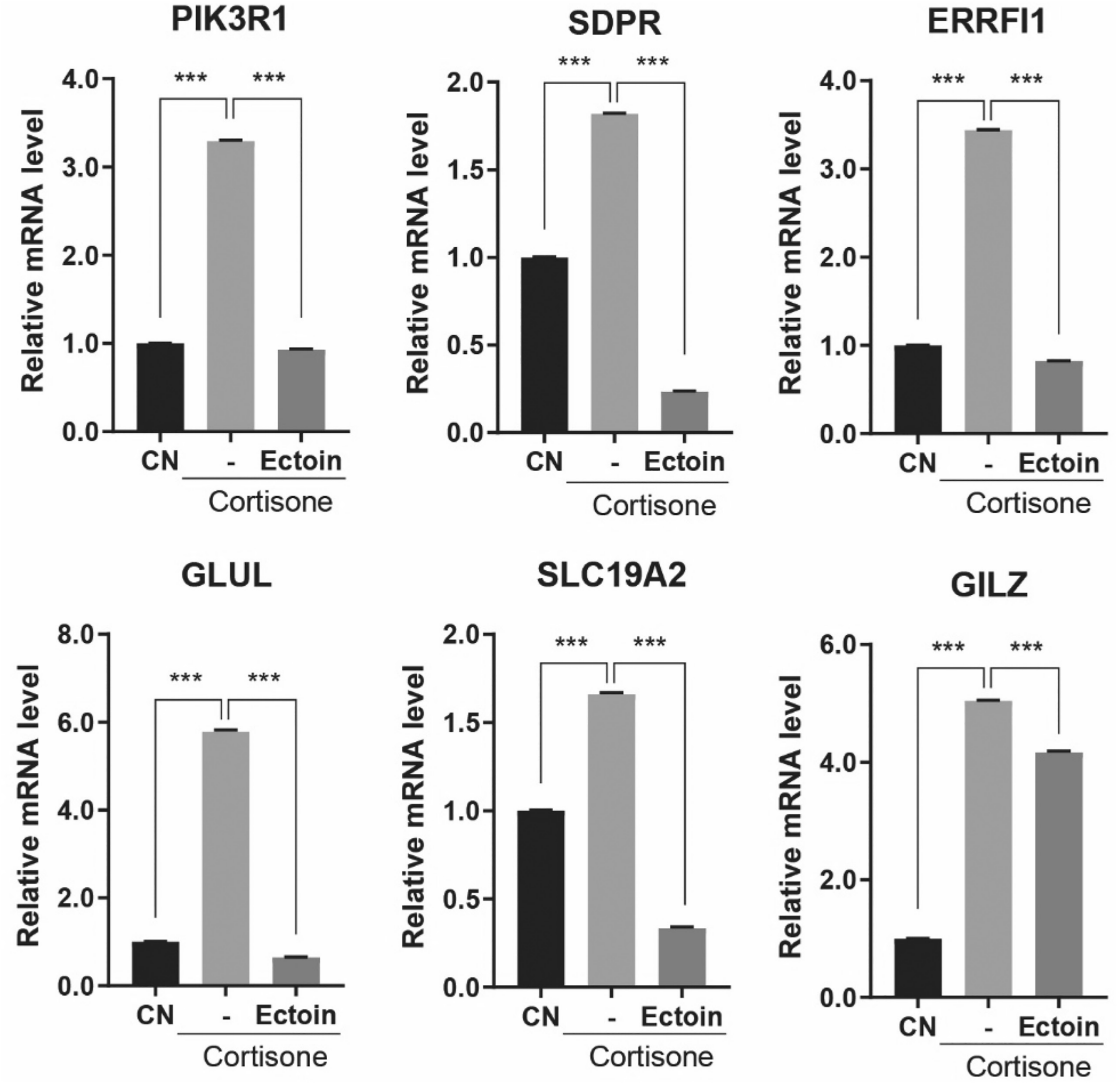
Ectoin suppresses cortisone‐induced GR target genes in NHDF. Treatment of the cells were either vehicle (labeled as “CTL”) or cortisone (100 μM) with or without Ectoin (0.5 mg/mL) for 12 h. Quantitative RT‐PCR was used to produce and evaluate total RNA samples. The mRNA expression of well‐known glucocorticoid‐responsive genes (PIK3R1, phosphoinositide‐3‐kinase regulatory subunit 1; SDPR, serum deprivation‐response protein; ERRFI1, ErbB receptor feedback inhibitor 1; GLUL, glutamate‐ammonia ligase; SLC19A2, solute carrier family 19 member 2; and GILZ, glucocorticoid‐induced leucine zipper) was evaluated. Data are reported as the mean ± SEM across the three experiments. The following is how statistical significance is expressed: ***0.001.

To explore the mechanism underlying the beneficial effects of Ectoin, we have identified six key GRs target gene candidates. This selection was based on the outcomes of prior target gene prediction analyses.^40^ We observed that the mRNA expression levels of these six genes—phosphoinositide‐3‐kinase regulatory subunit 1 (PIK3R1), serum deprivation‐response protein (SDPR), ErbB receptor feedback inhibitor 1 (ERRFI1), glutamate‐ammonia ligase (GLUL), solute carrier family 19 member 2 (SLC19A2), and glucocorticoid‐induced leucine zipper (GILZ)—were notably suppressed following treatment with Ectoin in cortisone‐exposed NHDF (Figure 6).

**FIGURE 7:**
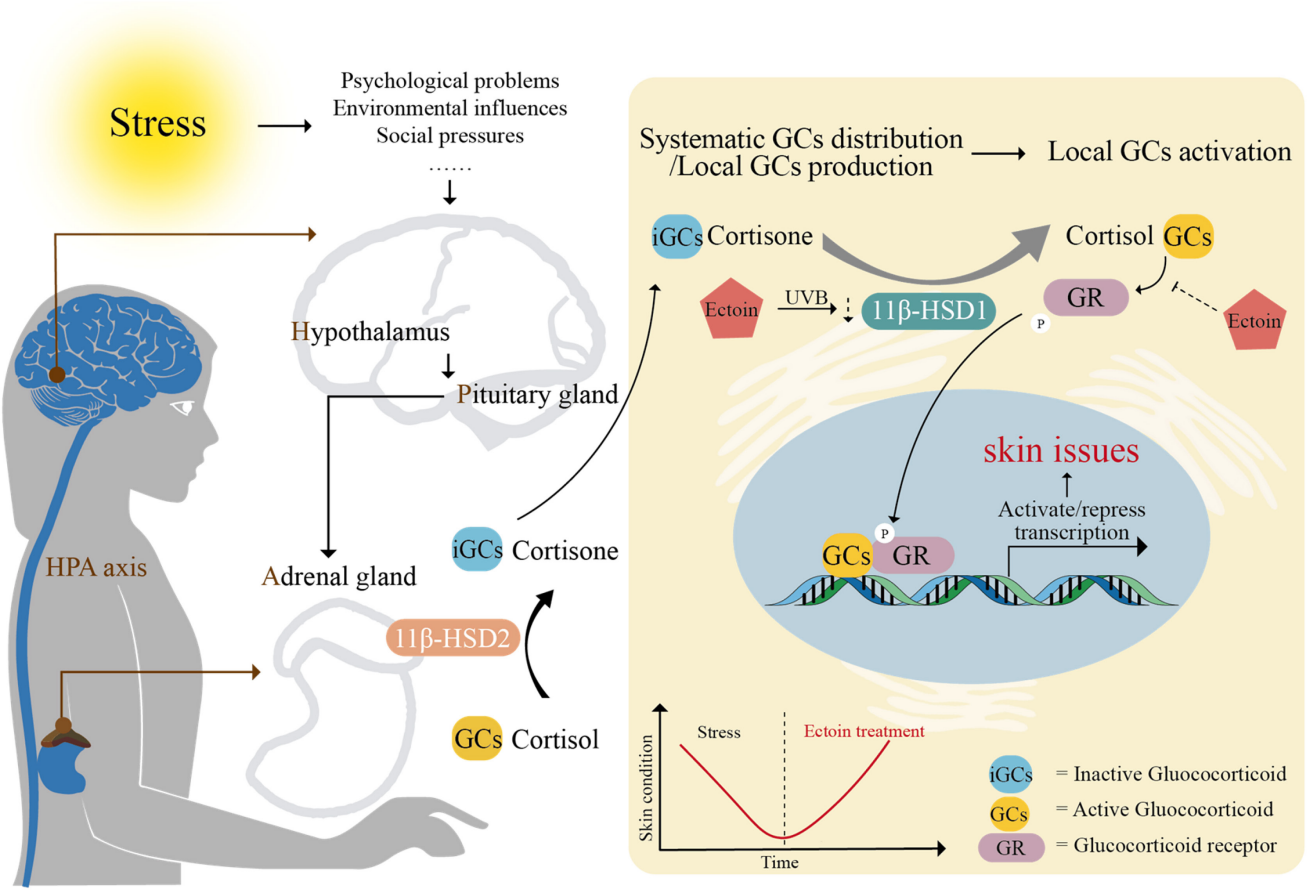
A suggested mechanism by which Ectoin attenuates cortisone‐induced skin issues.

In conclusion, the prevention of glucocorticoid binding to its receptors may underlie the efficacy of Ectoin in counteracting skin issues (Figure 7).

## 4 DISCUSSION

Emotional stress, physical injury, and disruptions in the circadian rhythm can all stimulate the hypothalamus to release corticotropin‐releasing hormone (CRH). In reaction to CRH, the anterior pituitary gland then releases adrenocorticotropic hormone (ACTH). The adrenal cortex secretes GCs in response to stimulation from ACTH, which has an immediate effect on the skin and other peripheral tissues.^12^ To imitate the typical process of physiological stress experienced by the body, we employed cortisone (a cortisol precursor) to replicate the stressed state of skin cells. To establish an appropriate cortisone dose, we evaluated the impact of varying cortisone levels on collagen production in human dermal fibroblasts (Figure S3). Ultimately, we selected a concentration of 100 μM as the optimal dose to mimic severe stress conditions commonly encountered in daily life, aligning with dosages referenced in the literature.^41^

The skin barrier is composed of a myriad of structures and elements, forming a complex “brick‐and‐mortar” architecture.^42^, ^43^, ^44^, ^45^, ^46^ Central to this model are the stratum corneum‐related structures, intercellular lipids, natural moisturizing factors (NMFs), and DEJ components. The cornified envelope, a vital aspect of the stratum corneum, is densely interwoven and predominantly composed of proteins such as LOR and INV, representing the “bricks” of the skin barrier.^45^, ^47^, ^48^ TJs among epidermal cells are deemed the primary regulators of cutaneous barrier mechanism.^49^, ^50^ Retaining skin barrier functionality can be realized by regulating the expression and connections of TJs, like CLDN1. The “mortar” consists of the main extracellular constituents of corneocytes: hydrophobic free ceramides, fatty acids, and cholesterol, which together provide a waterproof seal. NMFs present in the interstitial spaces of the epidermis and dermis, like HA, amino acids, pyrrolidone carboxylic acid, and uric acid, play a crucial role in hydrating the skin.^51^, ^52^ By binding to water molecules, these factors help maintain the skin's internal moisture, preserving its overall health and integrity. Moreover, another critical component of the skin barrier is DEJ.^53^, ^54^, ^55^ This structure, with its papillary projections that interdigitate with the epidermal ridges, reinforces the connection between the dermis and epidermis, thereby contributing to skin tautness and mitigating wrinkle formation. DEJ is mainly composed of a complex network with laminins and type IV collagen, set within a gel‐like matrix teeming with viscous polysaccharides. Its role is multifaceted: it increases the interface for substance exchange between the dermis and epidermis and serves vital osmotic and barrier functions. This research indicates that cortisone can disrupt function in skin cells at various layers, leading to a compromised skin barrier. The dysfunction is manifested by a reduction in the secretion of moisturizing factors, a decrease in the expression of structural proteins in epidermal layer, and damage to the integrity of TJs and DEJ. However, Ectoin treatment was observed to mitigate these effects, pointing to its potential in safeguarding the homeostasis of the skin barrier under conditions of stress.

GC‐induced skin aging is characterized by increased fragility, susceptibility to tearing and bruising, enlarged capillaries, and a compromised immune response. These effects are largely attributable to the impact of GCs on fibroblast proliferation and collagen metabolism.^16^ Research has established that 11β‐HSD1 is overexpressed in UV light‐exposed skin and naturally aged skin.^5^ Notably, selective intradermal injections of 11β‐HSD1 inhibitors have been observed to enhance dermal thickness and collagen density, underscoring the vital challenges to mitigate the negative influence of active cortisol on aging skin under stress.^22^, ^56^ Our study revealed that Ectoin could mitigate the negative impacts of cortisone on collagen secretion in NHDF at both genetic and protein levels, as well as diminish the breakdown of collagen by MMP‐1. Building on the considerable protective benefits Ectoin has shown against UVB‐induced 11β‐HSD1 overexpression, we hypothesize that Ectoin holds promise in mitigating the detrimental impacts of stress on aging skin, particularly in the context of light exposure.

GCs, secreted by adrenal glands, are able to circulate to various organs, including the skin. Additionally, the skin itself can generate cortisone through the steroidogenesis pathway, which initially impacts the dermis due to capillary distribution and then diffuses into the epidermis. Cortisone is activated to cortisol by 11β‐HSD1 expressed in the endoplasmic reticulum and then interacts with GRs. Two main GR types have been identified: the common GRα and the variant GRβ.^57^, ^58^, ^59^ Both arise from alternative splicing of the same GC receptor gene. While GRβ can bind to DNA, it does not interact with steroids and may inhibit steroid activity, disrupting GRα's DNA binding. Without GCs, GRα stays dormant in the cytoplasm, and is linked to heat shock proteins (HSPs), primarily HSP90 and HSP70, which serve as molecular chaperones.^60^ Specifically, the chaperone cycle for GR involves inactivation by HSP70, formation of an inactive GR–Hsp90–Hsp70–Hop ‘loading’ complex, transition to an active GR–Hsp90–p23’ maturation’ complex, and eventual release of GR.^61^, ^62^, ^63^ Upon entering to nucleus, GR attaches itself to glucocorticoid‐responsive elements at start sites of responsive genes, thereby intensifying their transcriptional activity and ensuing protein synthesis. This results in the production of proteins such as lipocortin‐1, adhesion molecules, endonucleases, neuropeptidases, and angiotensin‐converting enzymes, and leads to a spectrum of physiological and pharmacological effects.^18^, ^39^

In our studies, we've found that Ectoin acts as an antagonist that binds to GR, hindering GR activation. Ectoin, being a highly hydrophilic agent, forms a hydroelectric complex by surrounding itself with water molecules. This complex envelopes cells, enzymes, proteins, and other molecular structures in a protective hydrosheath.^64^ We propose that the highly hydrophilic properties of Ectoin obstruct the bonding of active GCs to their receptors, thereby safeguarding the skin from various stress issues, including stress‐induced aging and weakened barrier function. Furthermore, Ectoin has been shown to promote the expression of HSP70 and HSP70B in keratinocytes, influencing the inflammatory response.^65^ While there is no direct evidence of Ectoin affecting HSP90 expression, we hypothesize that Ectoin could regulate GR activation by modulating the expression of HSP70.

Stress acts as a double‐edged sword for the body. GCs are often recognized for their anti‐inflammatory properties by regulating various signaling pathways through GRs, such as the TLR‐mediated nuclear factor‐kappa B (NF‐κB) and activator protein‐1 (AP‐1), the mitogen‐activated protein kinase (MAPK) pathway, and interferon regulatory factors.^39^ However, GCs can also display pro‐inflammatory actions under acute stress.^19^ This is evident when GCs aggravate the peripheral immune response in patients with delayed‐type hypersensitivity who are receiving GC treatment.^66^ As a side effect of acute endogenous and exogenous stress, the role of GC signaling in mediating molecular actions in skin aging was preliminary described.^54^, ^67^, ^68^, ^69^, ^70^ In our investigation of the effects of Ectoin on cortisone‐treated human dermal fibroblasts, we determined that Ectoin downregulates six specific GR target genes. Among these, ERRFI1,^71^, ^72^ GILZ,^73^, ^74^, ^75^ and PIK3R1^76^ are noteworthy as inhibitors of the PI3K/AKT/mTOR signaling pathway, which is known to promote the synthesis of collagen and HA. Additionally, SDPR, a calcium‐independent phospholipid‐binding protein, acts as a substrate for protein kinase C, which upon activation can enhance the MAPK pathways.^77^, ^78^ These pathways can lead to the stimulation of transcription factors in human dermal fibroblasts, including NF‐κB and AP‐1, which is pivotal in various cellular processes. Excessive activation of GR induces the transcription and repression of a series of genes, ultimately leading to a fatigued appearance in the skin. We believe that there are far more than six major genes involved here. Future research focused on identifying GR target genes related to stress‐induced skin issues, as well as determining regulatory genes that have a beneficial impact on stressed skin with Ectoin treatment, will be a compelling focus.

## 5 CONCLUSION

Psychological distress and environmental shifts are increasingly acknowledged as key factors in stress‐related skin conditions. In our research, we uncovered the novel finding that Ectoin can alleviate skin issues triggered by active endogenous GCs through the suppression of GR signaling transduction. Ectoin also suppress the upregulation of 11β‐HSD1 induced by UVB exposure, consequently decreasing the levels of active endogenous GCs. This research highlights the potential utility of Ectoin in addressing skin aging and barrier dysfunction linked to various stressors. Additionally, our findings offer a deeper understanding of the potential molecular mechanisms through which Ectoin exerts its effects on human skin.

## CONFLICT OF INTEREST STATEMENT

The authors declared no potential conflicts of interest with respect to the research, author‐ship, and/or publication on of this article.

## ETHICS STATEMENT

An ethics statement was not required for this study type; no human or animal subjects were use.

## Supporting information


Data S1.

## Data Availability

The data that support the findings of this study are available from the corresponding author upon reasonable request.
