# Supplementary material for: Ectoin attenuates cortisone‐induced skin issues by suppression GR signaling and the UVB‐induced overexpression of 11β‐HSD1
Source: J Cosmet Dermatol. 2024 Sep 2;23(12):4303–14. doi: 10.1111/jocd.16516 (PMC11626367; doi:10.1111/jocd.16516)
Supplement: Supplementary file 1 — Data S1. [file JOCD-23--s001.docx]

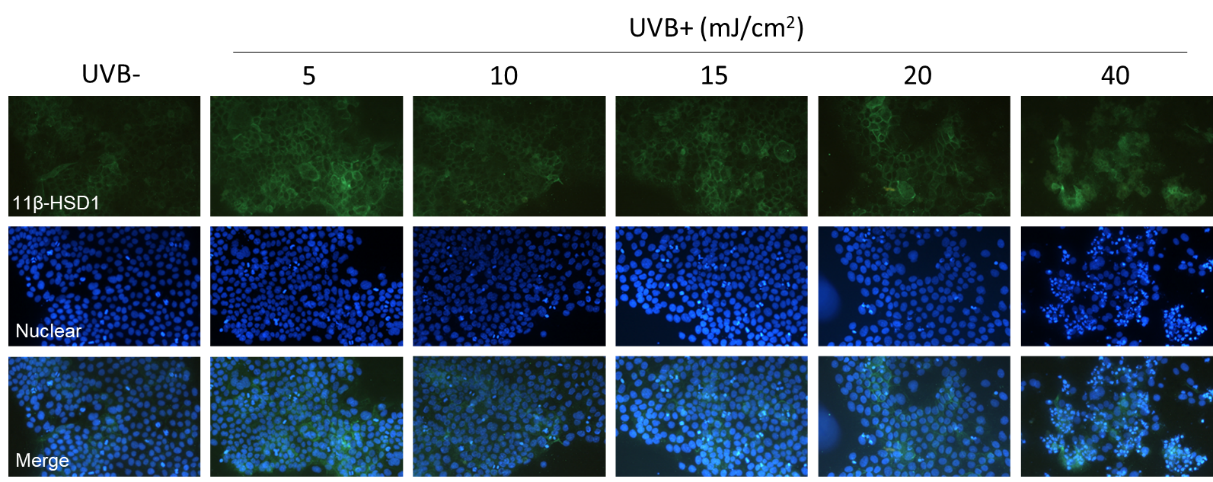


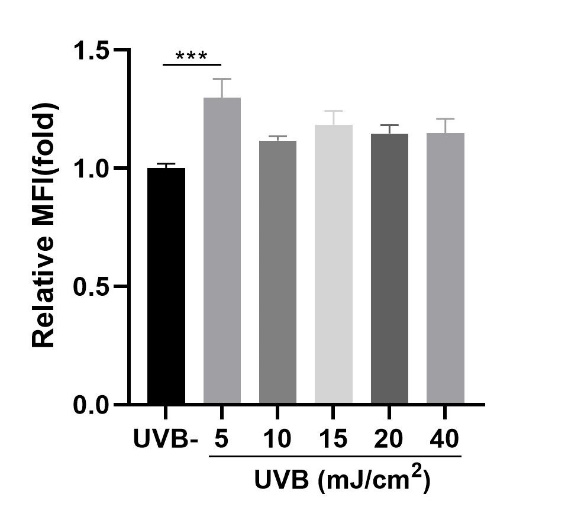


Supplementary Figure S1. Effect of UVB-irradiation on 11β-HSD1 expression in human immortalized epidermal (HaCaT) cells. The cells were plated in triplicate wells of 24-well plates and incubated over night. Subsequently, the cells were treated with UVB in a Serial doses (5, 10, 15,20,40 mJ/cm^2^), under a serum-free condition. Next, cells were cultured for another 72h and fixed in 4% formaldehyde, permeabilized with 0.1% triton X-100, and incubated with primary antibody over night at 4 ^o^C. Subsequently, the cells were washed 3 times with DPBS and then incubated with secondary antibodies correspoinding to species for 1.5h. Ultimately, the cells were examined, using the Leica DM2500LED+DMC6200. Values are shown as mean ± standard error of the mean (SEM) of three experiments. Statistical significance is expressed as follows: ns, not significant (p > 0.05). * p < 0.05, ** p < 0.01, *** p < 0.001.


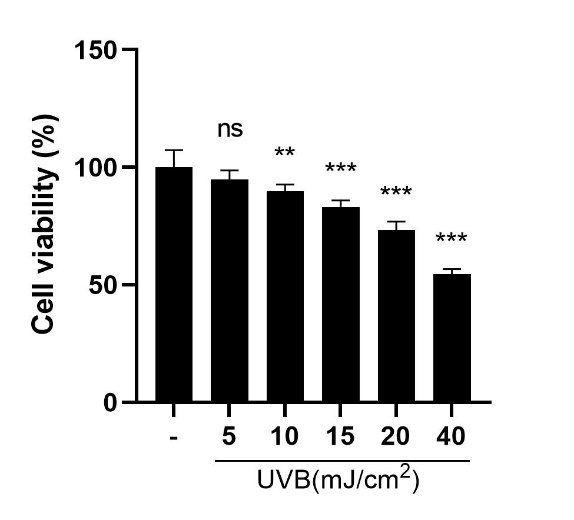


Supplementary Figure S2. Effects of UVB-irradiation on the viability of HaCaT cells. The cells were plated in triplicate wells of 96-well plates and incubated over night. Subsequently, the cells were treated with UVB in a Serial doses (5, 10, 15,20,40 mJ/cm^2^), under a serum-free condition. Next, cells were cultured for another 24h then the MTT reagent (0.5 mg/ml) was added to each well and the cells were incubated for 3 h. The medium was then discarded and the cells solubilized with DMSO. The absorbance was measured at a wavelength of 550 nm using a spectrophotometer.


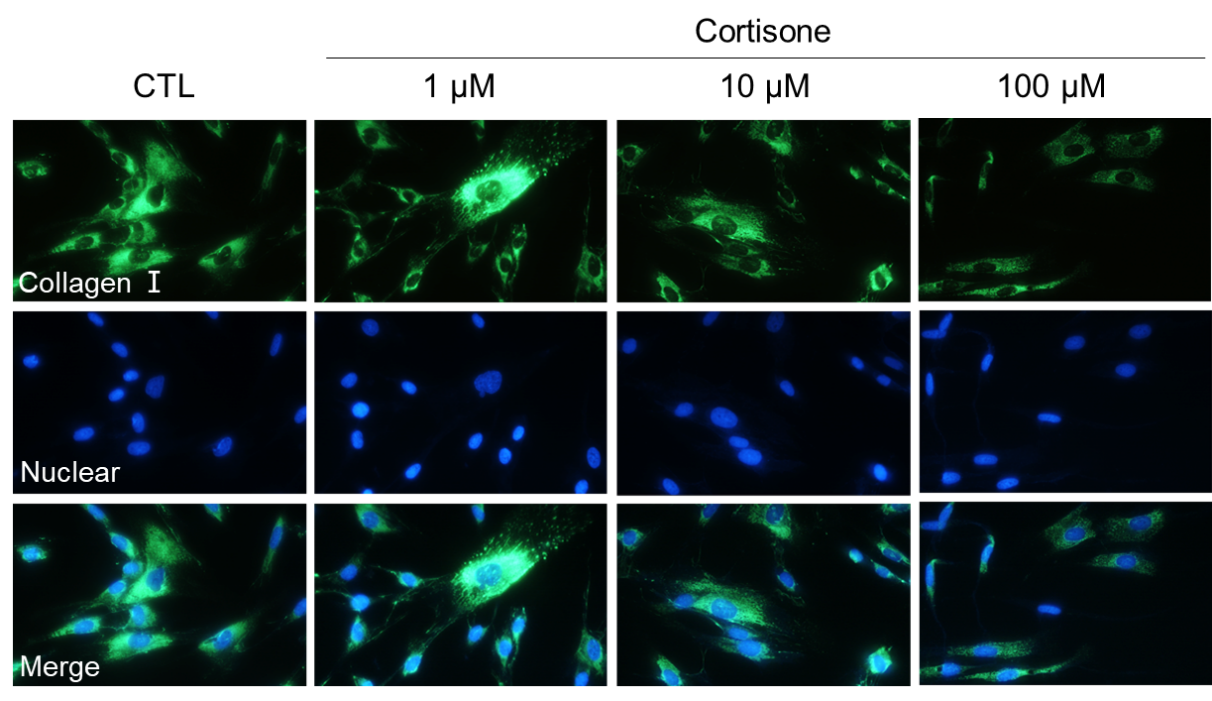


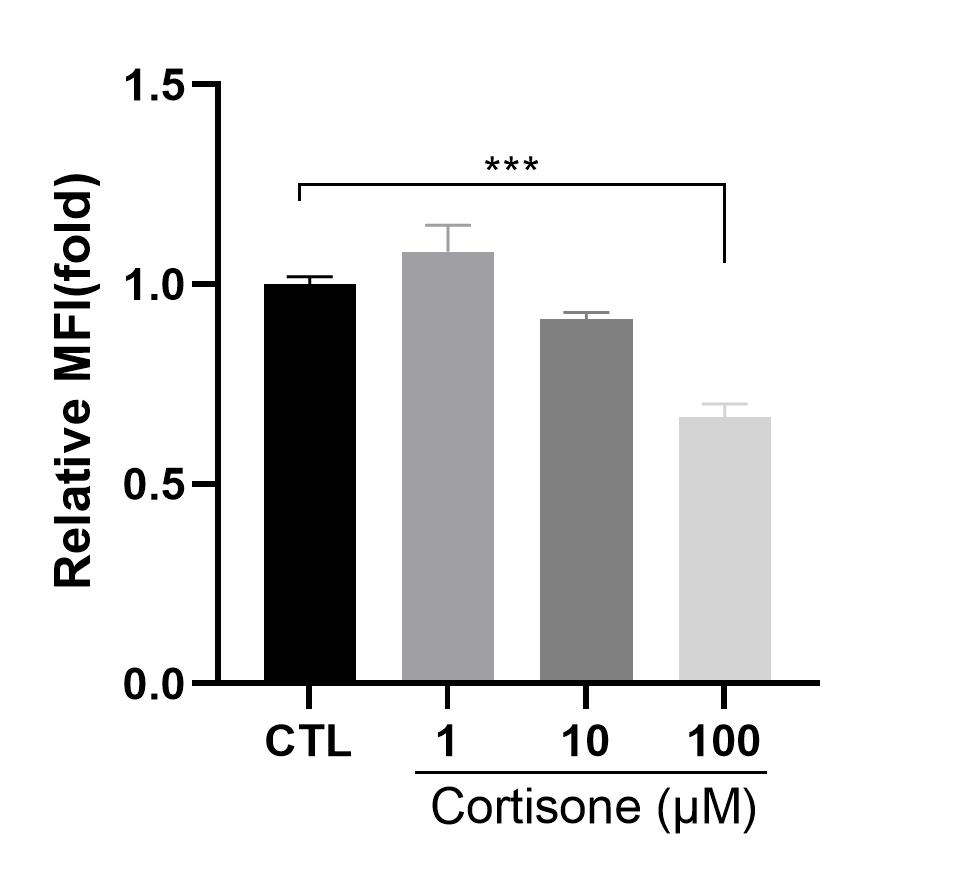


Supplementary Figure S3. Effect of cortisone on collagen type Ⅰ expression in human dermal fibroblasts. The cells were plated in triplicate wells of 24-well plates and incubated over night. Subsequently, the cells were treated with cortisone in a Serial doses (1, 10, 100 μM), under a serum-free condition. Next, cells were cultured for another 72h and fixed in 4% formaldehyde, permeabilized with 0.1% triton X-100, and incubated with primary antibody over night at 4 ^o^C. Subsequently, the cells were washed 3 times with DPBS and then incubated with secondary antibodies correspoinding to species for 1.5h. Ultimately, the cells were examined, using the Leica DM2500LED+DMC6200. Values are shown as mean ± standard error of the mean (SEM) of three experiments. Statistical significance is expressed as follows: ns, not significant (p > 0.05). * p < 0.05, ** p < 0.01, *** p < 0.001.

Supplementary Table S1. Primer sequences.

| Gene description | Sequence (5’→3′) |
| --- | --- |
| ERRFI1 | F: GGCCTCACAGGTTTGGAGATG  R: TTCATCGGAGCAGATTTGGAAG |
| SLC19A2 | F: AGCCAGACCGTCTCCTTGTA  R: TAGAGAGGGCCCACCACAC |
| PIK3R1 | F: AGCATTGGGACCTCACATTACACA  R: ACTGGAAACACAGTCCATGCACATA |
| GLUL | F: AGAAGAGCGGAGCGTGTGAG  R: CATGGTGGAAGGTGTTCTGGTC |
| GILZ | F: TCCTGTCTGAGCCCTGAAGAG  R: AGCCACTTACACCGCAGAAC |
| SDPR | F: AGTCACGGTGCTCACGCTCC  R: GTTGCTGGTGGAGGCCTGGT |
| GAPDH | F: TCTGGAAAGCTGTGGCGTGA  R: TACTTGGCAGGTTTCTCCAGG |
